# mol2chemfig, a tool for rendering chemical structures from molfile or SMILES format to LATE X code

**DOI:** 10.1186/1758-2946-4-24

**Published:** 2012-10-02

**Authors:** Eric K Brefo-Mensah, Michael Palmer

**Affiliations:** 1 Department of Chemistry, University of Waterloo, 200 University Avenue West, Waterloo, Ontario, N2L 3G1, Canada

**Keywords:** LATE X Chemfig, Molfile, SMILES, Molecular structures, Code generation

## Abstract

Displaying chemical structures in LATE X documents currently requires either hand-coding of the structures using one of several LATE X packages, or the inclusion of finished graphics files produced with an external drawing program. There is currently no software tool available to render the large number of structures available in molfile or SMILES format to LATE X source code. We here present mol2chemfig, a Python program that provides this capability. Its output is written in the syntax defined by the chemfig TE X package, which allows for the flexible and concise description of chemical structures and reaction mechanisms. The program is freely available both through a web interface and for local installation on the user’s computer. The code and accompanying documentation can be found at http://chimpsky.uwaterloo.ca/mol2chemfig.

## Background

**Figure 1:**
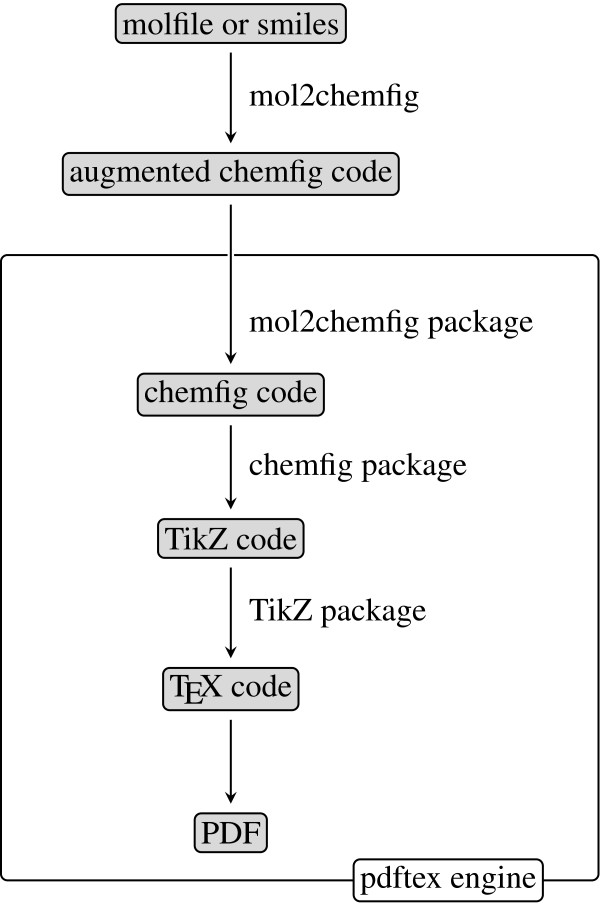
****mol2chemfig** processing flowchart.** Rendering molecular structures with mol2chemfig involves two separate executables, namely mol2chemfig itself (or, strictly speaking, the Python interpreter, which runs mol2chemfig) and a TE X engine such as pdftex. Processing inside TE X requires several packages, all of which will be loaded into LATE X by requiring the mol2chemfig package.

While TE X and LATE X provide excellent built-in support for mathematics and physics, the same cannot be said for chemistry. Several TE X and LATE X packages have been devised to address this lack of built-in support and to facilitate the hand-coding of chemical structures. Older examples of this approach are xymtex [1] and ppchTeX [2]. A recent development is chemfig [3], which in turn is built on top of the TiKZ general-purpose graphics package [4] (Figure 1). The syntax implemented by chemfig is remarkably concise and regular, which makes hand-coding of simple organic molecules effortless. The package offers many ways to customize the appearance of the rendered structures. It also facilitates the depiction of chemical reaction mechanisms, and it seems fair to say that chemfig sets a new standard for chemical illustrations in LATE X. Nevertheless, the hand-coding approach remains time-consuming with large molecules. The purpose of the mol2chemfig program described here is to remove this requirement by allowing for the generation of chemfig code from readily available chemical structure file formats.

The molfile [5] and the SMILES [6] data formats are widely used to represent molecule structures with or without atomic coordinates, respectively. The entries in the PubChem database [7] are available in both formats. Other chemical data formats can be converted to molfile or SMILES using converters such as openbabel [8], and most interactive chemical drawing programs can export these formats as well. We therefore chose molfile and SMILES as input formats for mol2chemfig.

## Implementation

mol2chemfig is written in Python version 2 [9]. It was tested only on Python 2.7 but uses no particular features of that version, and should therefore run on any recent Python 2.x installation. In addition to various modules from the standard library, it uses the indigo cheminformatics library and its accompanying Python API [10,11], which it relies on for parsing of molfile and SMILES input, addition or removal of hydrogen atoms, and the calculation of missing coordinates.

The program, which is used from the command line, and its required libraries can be installed on the user’s computer. Alternatively, a server installation of the program can be accessed through a web interface. As a third option, a command line-driven thin web client is available, which accepts input in the same way as the locally installed program but then hands it off to the server installation. The web interface is also implemented in Python. The thin client is implemented in Lua. Since TeXLive contains a Lua interpreter, it runs the thin client without installing any other software. MikTex should to the same, but the authors have not confirmed this. The thin client also transparently accesses the most up-to-date version of mol2chemfig.

The code in mol2chemfig is divided into several modules, whose functions are briefly outlined in Table 1. Additional information is contained within the doc strings and comments in the source code. Execution of the program involves the following major stages:

1. Using indigo, the molfile or SMILES input is read into the data structures defined by that library. If coordinates are missing (SMILES input) or the user has explicitly requested calculation of new ones, indigo is used to compute them.

**Table 1:** mol2chemfig code modules

**Module name(s)**	**Role**
processor	Accepts and validates user input from the command line or through the web; invokes indigo to parse input and supply missing coordinates; hands over to molecule
molecule	Generates tree representation of the molecule, applies options, renders molecule to chemfig code
chemfig_mappings	Supplies translations and auxiliary code for rendering the molecule tree to chemfig code
atom, bond	Supply auxiliary classes for molecule
common	Supplies auxiliary classes and global settings
options, optionparser	Define and process options

2. From the data structures populated by indigo, a tree representation of the molecule is built.

3. The tree is traversed and annotated in order to satisfy the user-selected options for molecule rotation, bond scaling and so forth.

4. The tree is rendered to chemfig code, which is returned.

The chemfig code generated by mol2chemfig uses several custom macros. These macros must be loaded by LATE X documents in order to execute the generated code; they are contained within a separate small LATE X package (mol2chemfig.sty) that also takes care of loading the chemfig package. The chemfig package, in turn, requires and loads the TiKZ package (Figure 1).

As of this writing, both TiKZ and chemfig are available in the two major TE X distributions (MikTeX and TeXLive). The custom LATE X code for mol2chemfig is included in this program’s download.

## Results and discussion

The use of the program and its features will here be illustrated with a few short examples; some more examples are contained in the documentation available through the program’s website, as well as in the Additional file 1 to this paper. While some basic elements of chemfig’s syntax will be briefly introduced, the latter will not be covered systematically. The chemfig package’s accompanying documentation is clearly written and thorough; reference [12] gives a brief but useful introduction.

### Basics of operation

The program is invoked from the command line. It takes exactly one argument, which by default is the name of a file containing a single molecule in molfile format. Output is written to stdout; output redirection will typically be used to write to a file instead. A miscellany of options is available to modify input and output. Invoking mol2chemfig -h or simply mol2chemfig will display the full list of options and their descriptions.

### Hand-written versus **mol2chemfig**-generated **chemfig** code

**Figure 2:**
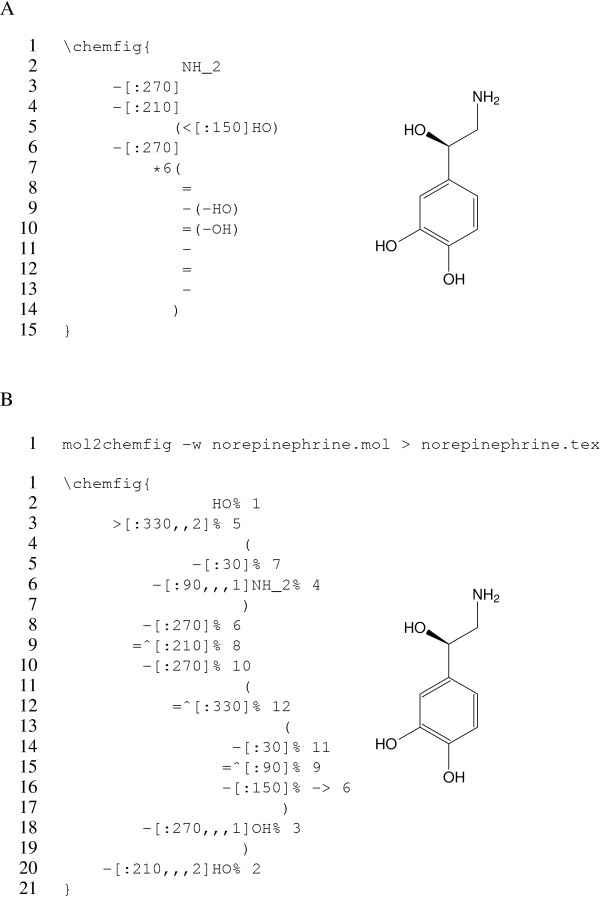
**Structure of norepinephrine, rendered with hand-written or **mol2chemfig**-generated **chemfig** code.** The code in **A** was hand-written and uses chemfig’s dedicated syntax for specifying rings (see lines 7–14). The code in **B** was generated with the mol2chemfig command shown at the top; it does not use chemfig’s ring syntax but instead treats the ring much like a regular branch. Each line of code specifies one bond; the number in the line-end comment specifies the atom that this bond connects to. While the code examples use line breaks and indentation for clarity, this is not required; whitespace is insignificant to chemfig.

Figure 2 depicts norepinephrine, rendered using either hand-crafted and mol2chemfig-generated code. The rendered result is very similar, although the double bonds in the ring are better proportioned in A. This difference arises from the use of chemfig’s syntax for rings in the hand-written version. The ⋆6(..) clause (spanning lines 7–14 in Figure 2A) declares a six-membered ring, and the - and = symbols within it denote the single and double bonds in the ring. Nested parentheses create branching bonds. Within the ring, specification of bond angles is not required, as they are inferred from the number of the ring atoms.

Outside of the ring, bond angles cannot be inferred and are specified explicitly between angular brackets. A preceding single colon denotes an absolute angle; an angle that is relative to the preceding bond can be specified with two colons, as in [::45]. Branches are again created by parentheses, as in line 5 of Figure 2A; this line also illustrates chemfig’s convention for specifying stereo bonds that point upwards. Since chemfig ignores whitespace, Figure 2A could also have been written as: ∖chemfigNH_2-[:270]-[:210](<[:150]HO)-[:270]⋆6(=-(-HO)=(-OH)-=-); this style might appeal to enthusiasts of the brainfuck language [13].

While elegant and effective for hand-coded molecules, chemfig’s syntax for rings is somewhat orthogonal to the tree syntax used with other parts of the molecule and thus is not implemented in mol2chemfig; therefore, the generated code in Figure 2B treats the ring much like the remainder of the molecule. By default, mol2chemfig uses one line for each bond and appends an end-of-line comment with the number of the atom that is reached by this bond; this number is the same as in the input if the latter is given in molfile format. If the number is prefixed with ->, as in line 16 in Figure 2B, this indicates that the bond closes a ring and points back to an atom that appeared in the output earlier.

**Figure 3:**
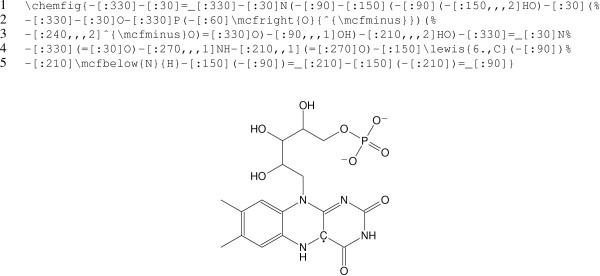
**Structure of FMNH.** The structure of FMNH (flavin mononucleotide hydride) contains charges and a radical, which are preserved during conversion with mol2chemfig. The chemfig code was generated using the --terse option, which removes whitespace and comments from the output.

In the generated code, line-end comments and dispensable whitespace can be suppressed by passing the -z or --terse option (see Figure 3 for an example). However, even with this option, generated code will tend to be more verbose than hand-crafted chemfig code and should not be taken as a model for how to write the latter.

A convenient method to include the code generated by mol2chemfig in a LATE X document is to load it from an external file with ∖input. Note, however, that ∖input cannot be used inside a ∖chemfig macro; therefore, the ∖chemfig macro must be part of the external file. The -w or --wrap-chemfig option used in Figure 2B assists with this by enclosing the generated code in a ∖chemfig macro.

### Charges and radicals

The molfile format can represent radicals and charges, and these are supported by mol2chemfig. Charges and radical electrons (as well as implicit hydrogens) are placed so as to minimize interference with bonds attached to the atom in question. Figure 3 shows the structure of FMNH as an example.

### Coordinate calculation and transformations

In 44A, the antitumor drug doxorubicin is rendered from coordinates obtained directly from PubChem; this is achieved using the -i pubchem or --input=pubchem option. Also used in this figure is the -y delete or --hydrogens=delete option, which converts all explicit hydrogens to implicit ones.

**Figure 4:**
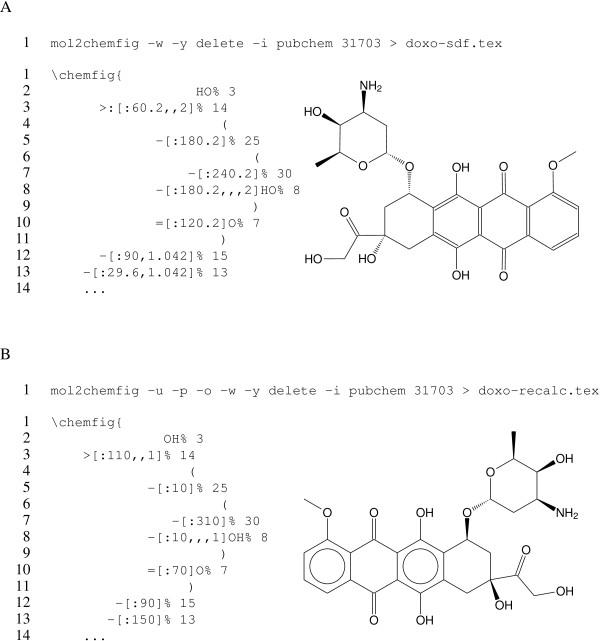
**Structure of doxorubicin.** The structure of doxorubicin, rendered from a PubChem record without **A** or with **B** recalculation of coordinates. The code examples in both **A** and **B** are truncated. See text for additional details.

In the rendered structure, the bond angles seem just a little off; this is confirmed by looking at the generated code, which shows angles that are close to, but not quite exactly the multiples of 30 degrees. Instead of fixing up all those angles manually, we can ask mol2chemfig to recalculate them for us with the -u or --recalculate-coordinates option; this is shown in Figure 4B. This example also illustrates the -p or --flip option to horizontally flip the molecule; other options allow vertical flipping and rotation. Finally, the -o or --aromatic-circles option renders aromatic rings with circles instead of discrete bonds.

Note that, in the recalculated structure, the orientations of some substituents are changed. These decisions are made by indigo, from which mol2chemfig adopts the coordinate calculation wholesale.

### Working with sub-molecules

**Figure 5:**
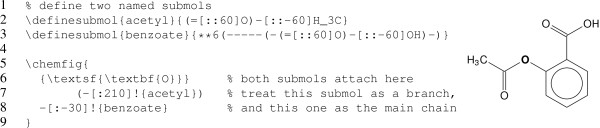
**Structure of aspirin, composed from two sub-molecules.** This hand-written example illustrates the use of chemfig’s submol mechanism. Two named sub-molecules are defined, which can then be referenced to compose the complete molecule.

The chemfig package allows us to define sub-molecules that we can reuse as parts of larger assemblies. Figure 5 illustrates this with a simple hand-coded example, in which the aspirin molecule is built using two sub-molecule definitions, named acetyl and benzoate, respectively.

**Figure 6:**
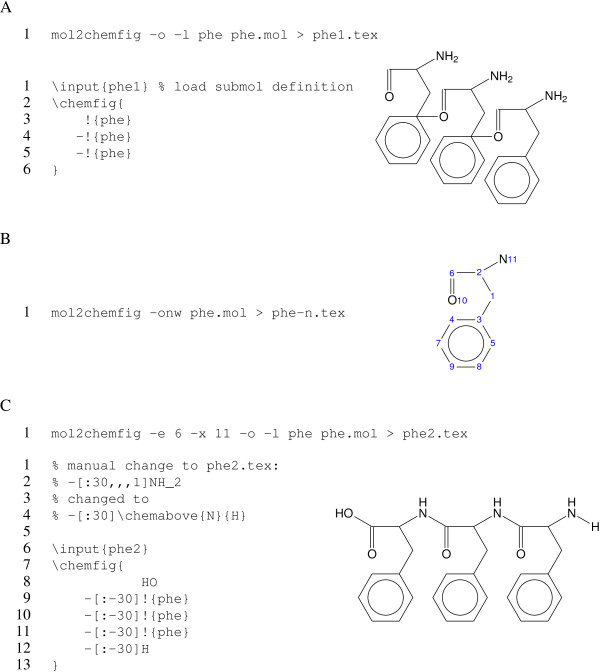
**Construction of a tripeptide from a **mol2chemfig**-generated aminoacyl residue.** The file containing the coordinates for a phenylalanyl residue was rendered to a ∖submol definition, and three copies of the latter were concatenated. In **A**, mol2chemfig was allowed to arbitrarily pick the first and last atoms of the sub-molecule’s main chain, which causes the connecting bonds to be misplaced. In **B**, the atom numbers of the molfile were displayed using the -n or --atom-numbers option. In **C**, atoms 6 and 11 were specified as the main chain entry and exit points, respectively; this causes the connecting bonds to be placed as intended. In the generated code, the amino group was manually adjusted.

Let us assume we want to build the tripeptide shown in 6 Figure 6C. We can use the -l phe or --submol-name=phe option to render the phenylalanyl-residue to a named sub-molecule definition (using the name phe). However, a naive first attempt fails (Figure 6A), since it connects the wrong atoms between successive sub-molecules.

The submol mechanism operates essentially through string substitution; therefore, subsequent sub-molecules are simply connected across the last and first atoms of their respective main chains. In order to place those connecting bonds correctly, we thus need to take control of the entry and exit atoms for the sub-molecules. To find the correct ones, we can let mol2chemfig print the atom numbers, as illustrated in Figure 6B. Setting atoms 6 and 11 as entry and exit atoms, respectively, then produces the structure shown in Figure 6C.

Note that, in the sub-molecule definition generated for Figure 6C, the primary amino group was manually changed to a secondary one. Generally speaking, while basic usage of mol2chemfig does not require familiarity with chemfig’s syntax, the ability to manually touch up the generated code will notably increase the usefulness of this program. The chemfig package offers a plethora of settings for bond lengths, colors and patterns as well as font sizes and shapes that allow the user to tweak the appearance of the rendered structures. It also provides facilities to depict reaction mechanisms and schemes; structures generated with mol2chemfig can be manually modified and incorporated into such schemes.

## Conclusion

The mol2chemfig program introduced here allows the conversion of molecules specified in molfile or SMILES format to the TE X-compatible format defined by the chemfig package. The generated code can be included in documents as is, or can be edited and integrated into larger chemfig graphics. We hope the program will be useful for authors who wish to illustrate the structures of organic molecules and reactions in LATE X documents.

## Availability and requirements

**Project Name:** mol2chemfig**Project home page:** http://chimpsky.uwaterloo.ca/mol2chemfig/**Operating system(s):** Linux, Windows, Mac**Programming language:** Python 2.7**Other requirements:** For full version: Python 2.7, the indigo toolkit and its prerequisite libraries; for thin client: a Lua interpreter. The LuaTeX binary that is available through TeXLive or MikTeX satisfies this requirement. (The manual installation of indigo is described at https://github.com/ggasoftware/indigo/blob/master/README.txt; binary packages are available for several Linux distributions.)**Any Restrictions to use by non-academics:** None. The code is freely available under the LATE X public license.

The locally installable full version and the thin web client are packaged and available for download from the project’s website. The server setup that is used by both the web interface and the web client is not routinely available, but the required code and setup instructions will be shared upon request.

## Competing interests

The authors declare that they have no competing interests.

## Author’s contributions

EB-M: code implementation and testing, preparation of manuscript; MP: code implementation, preparation of manuscript. Both authors have read and approved the manuscript.

## Supplementary Material

Additional file 1 mol2chemfig sample LATE X document. Click here for file
