# Supplementary material for: mol2chemfig, a tool for rendering chemical structures from molfile or SMILES format to LATE X code
Source: J Cheminform. 2012 Oct 2;4:24. doi: 10.1186/1758-2946-4-24 (PMC3551648; doi:10.1186/1758-2946-4-24)
Supplement: Additional file 1 — mol2chemfig sample LATE X document. [file 1758-2946-4-24-S1.zip › latex/sample.pdf]

Sample LaTeX document for `mol2chemfig`

This sample document illustrates the use of `mol2chemfig` in conjunction with the `chemfig` package. This document should compile as is on any system that has a working installation of the `chemfig` package. A local installation of `mol2chemfig` is not required to compile it, but it *is* required to run the example commands that were used to generate the code. As an alternative to local installation, you can use the web interface at `chimpsky.uwaterloo.ca/mol2chemfig`.

The structure of penicillin G

The `chemfig` code contained in the file `penicilling.tex` was generated from the molfile with the command:

```
mol2chemfig -w penicilling.mol > penicilling.tex
```

With `chemfig`'s default settings, the structure comes out as follows:

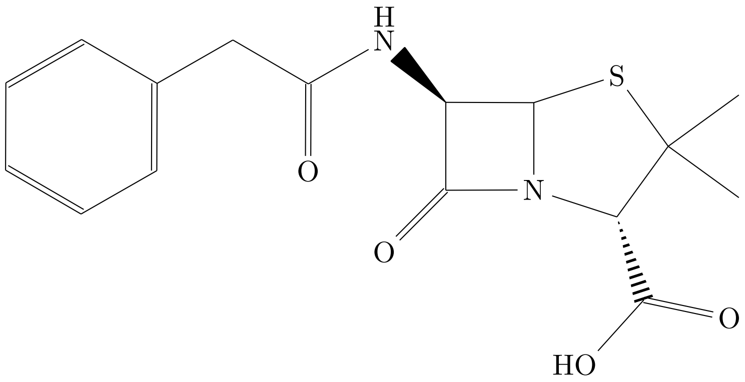

This is a little bit out of proportion to the surrounding text. We can easily adjust the appearance by tweaking some of the settings provided by `chemfig`:

```
% bond styling
\setcrambond{2.5pt}{0.4pt}{1.0pt}
\setbondoffset{1pt}
\setdoublesep{2pt}
\setatomsep{16pt}
% print atoms with smaller font and in sans-serif
\renewcommand{\printatom}[1]%
{\fontsize{8pt}{10pt}\selectfont{\ensuremath{\mathsf{#1}}}}%
```

After these settings have been adjusted, the same structure now appears as follows:

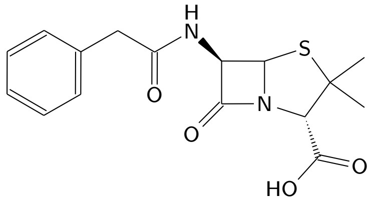

We will leave these settings in effect for the remainder of this document.

Incorporating rendered structures into composite graphics

The `mol2chemfig` package loads `chemfig`, which in turn loads the general purpose graphics package `TikZ`. Through the latter package, we have access to the `tikzpicture` environment. Here is a `tikzpicture` that depicts two resonance structures of alanine bound to pyridoxal phosphate:

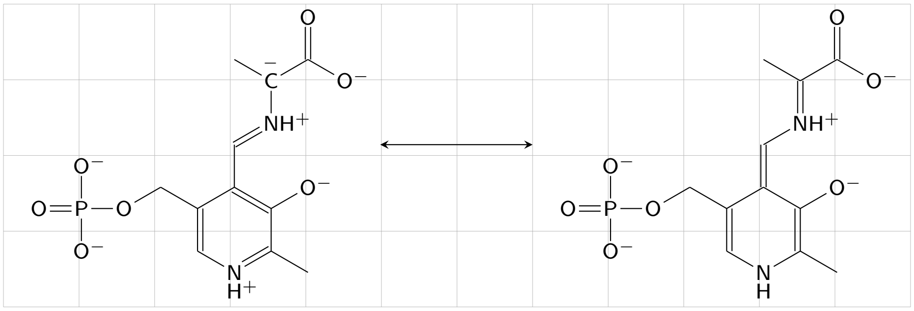

Note that the `\input` macro cannot be used inside a `\node` in the `tikzpicture` environment. As a workaround, the two structures were rendered as `\submol` definitions, which were `\input` outside the `tikzpicture` environment and then referenced from within the `\node` macros.

Of course, to be complete and valid, the scheme above should also include arrows that indicate the electron movements underlying the resonance effect. This is possible in `chemfig` but will in this case require manual annotation of the generated code. So that we can find our way through the code, we can first render the structure to be annotated with atom numbers and display it:

```
mol2chemfig -wn plp2.mol > plpn.tex
```

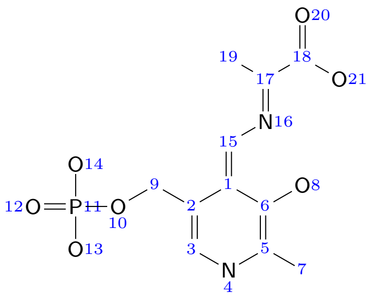

This tells us that we need to draw electron movement arrows from nitrogen 4 to the adjacent bond to carbon 3, from bond 2→3 to bond 1→2, and from bond 1→15 to bond 15→16. These atoms and bonds need to be given handles for the arrows to attach to. The listing below shows the edited version; the manual changes are highlighted.

```
\definesubmol{plp3}{
            % 7
     -[:150]% 5
     % add an electron pair and a named handle to atom 4
     % -[:210]\mcfbelow{N}{H}% 4 - original line
     -[:210]@{atom4}\mcfbelow{\lewis{2,N}}{H}% 4
     -[@{bond3to4}:150]% 3 add named handle to bond
     =_[@{bond2to3}:90]% 2
                (
         -[:150]% 9
         -[:210]O% 10
         -[:180]P% 11
                    (
             -[:270]\mcfright{O}{^{\mcfminus}}% 13
                    )
                    (
              -[:90]\mcfright{O}{^{\mcfminus}}% 14
                    )
         =[:180]O% 12
                )
      -[@{bond1to2}:30]% 1
                (
          =[@{bond1to15}:90]% 15
      -[@{bond15to16}:30,,,1]NH^{\mcfplus}% 16
        =[:90,,1]% 17
                    (
              -[:150]% 19
                    )
          -[:30]% 18
                    (
              -[:330]\mcfright{O}{^{\mcfminus}}% 21
                    )
          =[:90]O% 20
                )
     -[:330]% 6
                (
          -[:30]\mcfright{O}{^{\mcfminus}}% 8
                )
    =_[:270]% -> 5
}
```

We can now reference the handles to attach the electron movement arrows. Note that, for this to work, the document has to be processed *twice* by pdflatex, since the commands internally use a PDF overlay mechanism; otherwise, the arrows may be misplaced.

The `\mcfpush` macro that is used to place the arrows is defined in the `mol2chemfig` package and is explained in the source code of this document; it is a convenience wrapper around the `\chemmove` command provided by `chemfig`.

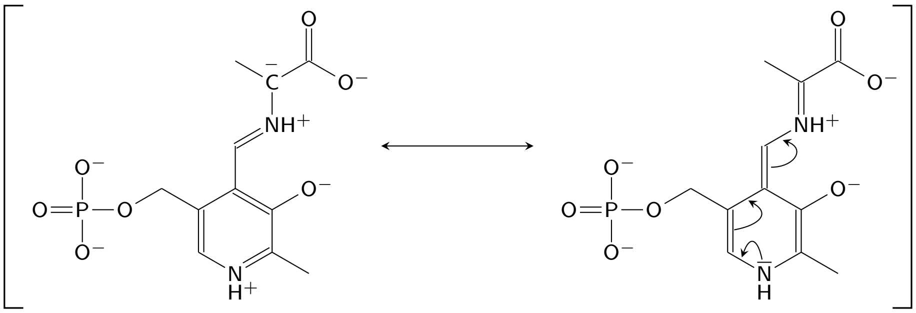

Invoking mol2chemfig from within LaTeX

Using the -shell-escape option on Linux or its equivalents on other systems, LaTeX can execute shell commands, capture the output and insert it directly into the document. We can use this with mol2chemfig. If you have mol2chemfig working and LaTeX properly configured, the following command will insert the structure of FMNH directly into your document, without creating a separate file:

```
\mcfinput|"mol2chemfig -w fmn.mol"
```

Note, however, that with large documents and numerous formulas the overhead of running mol2chemfig on each formula each time will add up.
